# Supplementary material for: Facile Gold-Nanoparticle Boosted Graphene Sensor Fabrication Enhanced Biochemical Signal Detection
Source: Nanomaterials (Basel). 2022 Apr 12;12(8):1327. doi: 10.3390/nano12081327 (PMC9033081; doi:10.3390/nano12081327)
Supplement: Supplementary file 1 [file nanomaterials-12-01327-s001.zip › nanomaterials-1667437-supplementary.pdf]

nanomaterials

MDPI

Supplementary Material

Facile Gold-Nanoparticle Boosted Graphene Sensor Fabrication Enhanced Biochemical Signal Detection

Shuaishuai Meng [1], Li Wang [1], Xixi Ji [2], Jie Yu [2], Xing Ma [1], Jiaheng Zhang [1], Weiwei Zhao [1], Hongjun Ji [1], Mingyu Li [1] and Huanhuan Feng [1,*]

[1] Sauvage Laboratory for Smart Materials, Flexible Printed Electronic Technology Center, School of Materials Science and Engineering, Harbin Institute of Technology (Shenzhen), Shenzhen 518055, China; m15052689951@163.com (S.M.); 18s154783@stu.hit.cn (L.W.); maxing@hit.edu.cn (X.M.); zhangjiaheng@hit.edu.cn (J.Z.); wzhao@hit.edu.cn (W.Z.); jhj7005@hit.edu.cn (H.J.); myli@hit.edu.cn (M.L.)
[2] School of Materials Science and Engineering, Harbin Institute of Technology (Shenzhen), Shenzhen 518055, China; jixixi201408@163.com (X.J.); jyu@hit.edu.cn (J.Y.)
* Correspondence: fenghuanhuan@hit.edu.cn; Tel./Fax: +86-755-86148426

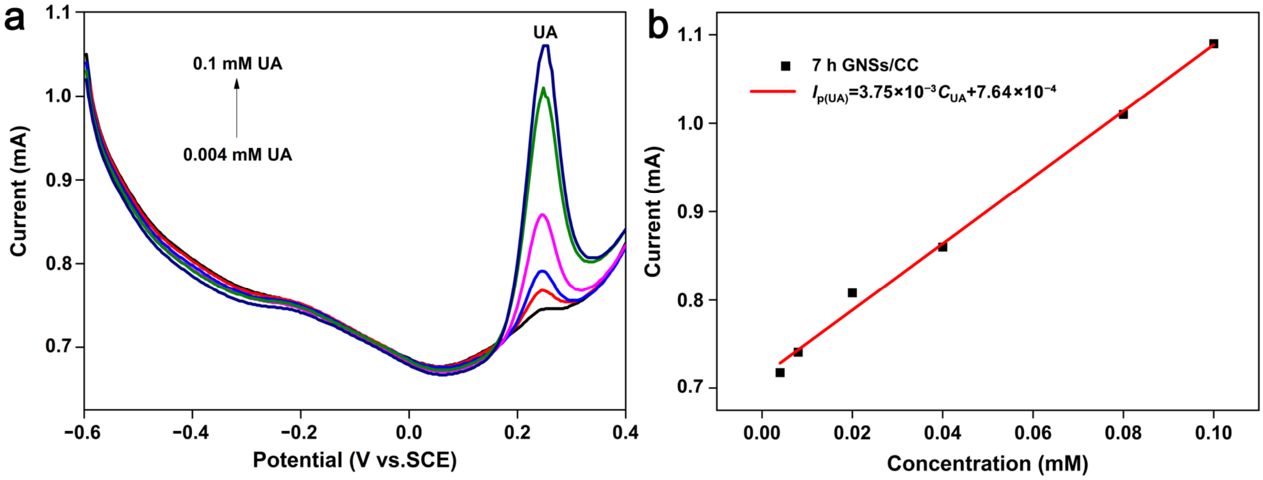

Figure S1. (**a**) DPV curve of UA detected by 7 h GNSs/CC electrode. (**b**) Fitting curve of current and concentration of UA detected by 7 h GNSs/CC electrode.

Figure S1a shows the PDV curve of the 7 h GNSs/CC electrode in 0.1 M PBS solution at a rate of 50 mV s^{-1} from –0.6 to 0.4 V of UA at different concentrations. The oxidation peak potential of UA was 264 mV. When the concentration of UA increased, the oxidation peak potential position almost did not move. Fig. S1b shows the fitting curve of the corresponding current and concentration. With an increase in UA concentration, the corresponding peak current increased gradually and the peak current was correlated linearly with the corresponding concentration. The linear equation was $I_P(\mu A) = 3750\ C_{UA}+763.6$ ($R^2 = 0.994$) and the linear concentration range was 0.004–0.1 mM. The sensitivity of the 7-h GNSs/CC electrode to UA was 1875 μA mM^{-1} cm^{-2}, and the minimum detection concentration was 0.001 mM.
